# Identification of the biological processes, immune cell landscape, and hub genes shared by acute anaphylaxis and ST-segment elevation myocardial infarction

**DOI:** 10.3389/fphar.2023.1211332

**Published:** 2023-07-04

**Authors:** Zekun Peng, Hong Chen, Miao Wang

**Affiliations:** ^1^ State Key Laboratory of Cardiovascular Disease, Fuwai Hospital, National Center for Cardiovascular Diseases, Chinese Academy of Medical Sciences and Peking Union Medical College, Beijing, China; ^2^ Clinical Pharmacology Center, Fuwai Hospital, National Center for Cardiovascular Diseases, Chinese Academy of Medical Sciences and Peking Union Medical College, Beijing, China

**Keywords:** anaphylaxis, STEMI, immune response, inflammation, hub gene

## Abstract

**Background:** Patients with anaphylaxis are at risk for ST-segment elevation myocardial infarction (STEMI). However, the pathological links between anaphylaxis and STEMI remain unclear. Here, we aimed to explore shared biological processes, immune effector cells, and hub genes of anaphylaxis and STEMI.

**Methods:** Gene expression data for anaphylactic (GSE69063) and STEMI (GSE60993) patients with corresponding healthy controls were pooled from the Gene Expression Omnibus database. Differential expression analysis, enrichment analysis, and CIBERSORT were used to reveal transcriptomic signatures and immune infiltration profiles of anaphylaxis and STEMI, respectively. Based on common differentially expressed genes (DEGs), Gene Ontology analysis, cytoHubba algorithms, and correlation analyses were performed to identify biological processes, hub genes, and hub gene-related immune cells shared by anaphylaxis and STEMI. The robustness of hub genes was assessed in external anaphylactic (GSE47655) and STEMI (GSE61144) datasets. Furthermore, a murine model of anaphylaxis complicated STEMI was established to verify hub gene expressions. The logistic regression analysis was used to evaluate the diagnostic efficiency of hub genes.

**Results:** 265 anaphylaxis-related DEGs were identified, which were associated with immune-inflammatory responses. 237 STEMI-related DEGs were screened, which were involved in innate immune response and myeloid leukocyte activation. M0 macrophages and dendritic cells were markedly higher in both anaphylactic and STEMI samples compared with healthy controls, while CD4^+^ naïve T cells and CD8^+^ T cells were significantly lower. Enrichment analysis of 33 common DEGs illustrated shared biological processes of anaphylaxis and STEMI, including cytokine-mediated signaling pathway, response to reactive oxygen species, and positive regulation of defense response. Six hub genes were identified, and their expression levels were positively correlated with M0 macrophage abundance and negatively correlated with CD4^+^ naïve T cell abundance. In external anaphylactic and STEMI samples, five hub genes (IL1R2, FOS, MMP9, DUSP1, CLEC4D) were confirmed to be markedly upregulated. Moreover, experimentally induced anaphylactic mice developed impaired heart function featuring STEMI and significantly increased expression of the five hub genes. DUSP1 and CLEC4D were screened as blood diagnostic biomarkers of anaphylaxis and STEMI based on the logistic regression analysis.

**Conclusion:** Anaphylaxis and STEMI share the biological processes of inflammation and defense responses. Macrophages, dendritic cells, CD8^+^ T cells, and CD4^+^ naïve T cells constitute an immune cell population that acts in both anaphylaxis and STEMI. Hub genes (DUSP1 and CLEC4D) identified here provide candidate genes for diagnosis, prognosis, and therapeutic targeting of STEMI in anaphylactic patients.

## 1 Introduction

Anaphylaxis is a severe hypersensitivity reaction that occurs rapidly after allergen irritation (medications, foods, insect venom) to sensitized individuals. It typically manifests with severe pathophysiological symptoms, such as respiratory distress, angioedema, and myocardial depression (LoVerde et al., 2018; Cardona et al., 2020). The prevalence of anaphylaxis among U.S. residents is estimated at 1.6%–5.1% (Wood et al., 2014), and the mortality rate for hospitalized patients is 0.5%–1% (Turner et al., 2020). In most cases, anaphylaxis is initiated by the allergen-IgE/IgG complex-induced activation of immune effector cells, followed by the release of inflammatory mediators that cause vascular hyperpermeability, bronchoconstriction, and airway edema (Finkelman et al., 2016; LoVerde et al., 2018).

Anaphylactic reactions may trigger adverse cardiovascular events (Abdelghany et al., 2017), such as ST-segment elevation myocardial infarction (STEMI) (Engheta et al., 2021), a severe form of heart attack with high mortality rate. A nationwide epidemiological study in the United States reported that among 235,420 patients hospitalized for allergy, hypersensitivity, or anaphylaxis, 0.2% of patients experienced STEMI (Desai et al., 2019). Acute STEMI following anaphylaxis is associated with allergic mediators-induced coronary spasms, plaque erosion/rupture, or stent thrombosis (Li et al., 2018; Sakaue et al., 2020; Yamamoto et al., 2022). Inflammatory responses elicited by mast cell-released vasoactive substances appears to be involved in this process and aggravate myocardial injury (Galli and Tsai, 2012; Abdelghany et al., 2017; Li et al., 2018; Yamamoto et al., 2022). Clinically, anaphylaxis complicated with STEMI is one of the most serious emergencies without effective predictors and medications. Due to severe cardiac ischemia and output depression, anaphylaxis-related STEMI might eventually progress to cardiovascular collapse and cause a fatal outcome. However, key molecules and immune cell subsets that drive the development of this complication have not been fully characterized.

Transcriptomic analysis has become an emerging approach for uncovering the complex pathophysiological processes in anaphylaxis and STEMI (Rung and Brazma, 2013; Xu and Yang, 2021; Rijavec et al., 2022). The common biological processes and signal transduction pathways of anaphylaxis and STMEI might indicate the underlying mechanisms for the coexistence of these two diseases. In particular, due to the ease of access and preservation of peripheral blood samples, in-depth analysis of gene expression profiles and screening hub genes in diseased specimens may allow identification of the whole blood gene signature shared by anaphylaxis and STEMI, facilitating precise diagnosis, prediction and drug discovery for anaphylaxis complicated STEMI.

Herein, we analyzed whole blood transcriptomic datasets of anaphylaxis and STEMI patients and identified the pathological processes, immune effector cell subsets, and hub genes associated with both anaphylaxis and STEMI, and we also established a mouse model of anaphylaxis complicated with STEMI and further validated the hub genes. The genomic signatures identified here may provide novel insights into the pathogenesis of anaphylaxis-related STEMI.

## 2 Materials and methods

### 2.1 Microarray data collection

The microarray data of anaphylactic patients, STEMI patients, and corresponding healthy controls were pooled from the public GEO database (https://www.ncbi.nlm.nih.gov/geo/). Datasets that satisfied the following inclusion criteria were selected: (1) gene expression profiles were based on human specimens; (2) all samples were obtained from peripheral blood; (3) the datasets included disease cases and healthy controls. GSE69063 (anaphylaxis) and GSE60993 (STEMI) datasets that meet the above criteria were used in the present study. GSE69063 contains 17 anaphylactic patients and 10 healthy controls whose blood specimens were collected 1 h after arriving at the emergency department. GSE60993 dataset consists of 7 STEMI patients and 7 healthy individuals, and peripheral blood from STEMI patients was obtained within 4 h after the attack of chest pain. Details of these datasets were summarized in Supplementary Table S1.

### 2.2 DEG screening

DEGs between case and control groups were screened by GEO2R (an official web application in NCBI that helps analyze GEO data, www.ncbi.nlm.nih.gov/geo/ge2r) (Barrett et al., 2013), with screening criteria set as |logFC (fold change)|>1 and adjusted p.value < 0.05. Probe sets without corresponding gene symbols were removed and genes with multiple probe sets were averaged. The volcano map was drawn with ggplot2 R package (https://ggplot2.tidyverse.org). TBtools software was applied to draw heatmaps to visualize the differential gene expression profiles between case and control groups (Chen et al., 2020).

### 2.3 Enrichment analysis of DEGs

Gene Ontology (GO) enrichment analysis and Kyoto Encyclopedia of Genes and Genomes (KEGG) pathway analysis were performed with the clusterProfiler package (Yu et al., 2012). The top 20 terms of biological process (BP) and top 5 KEGG terms were visualized with bubble charts by ggplot2 R package.

### 2.4 Analysis of immune cell distribution

The composition and distribution of immune cells in anaphylactic samples (GSE69063), STEMI samples (GSE60993), and normal samples were evaluated by CIBERSORTx (https://cibersortx.stanford.edu/) (Newman et al., 2015), which is based on a set of feature gene expression data from 22 cell subtypes (LM22 dataset). The fraction of these cell populations in diseased and healthy groups were compared using the ggpubr R package (Wilcoxon test) (https://CRAN.R-project.org/package=ggpubr).

### 2.5 Protein-protein interaction network construction and hub gene identification

STRING database was applied to predict PPI (https://cn.string-db.org/) (Franceschini et al., 2013) and a combined score >0.4 was considered statistically significant. Cytoscape software (Shannon et al., 2003) was used to visualize PPI networks. DEGs in the intersection of six algorithms (DMNC, Stress, MCC, Degree, Closeness, and Radiality) were determined as hub genes. The co-expression and co-localization network of hub genes was constructed and visualized by GeneMANIA (http://www.genemania.org/) (Warde-Farley et al., 2010).

### 2.6 Verification of hub gene expression in GSE47655 and GSE61144

Hub gene expression was confirmed in the GSE47655 (anaphylaxis) and GSE61144 (STEMI) datasets. GSE47655 contains 6 anaphylactic patients and 6 healthy controls. GSE60993 dataset consists of 7 STEMI patients and 10 healthy subjects. All samples from GSE47655 and GSE61144 were obtained from peripheral blood. Details of these datasets were shown in Supplementary Table S1. The differences in mRNA expression of hub genes between the diseased and healthy groups were compared using Student’s t-test and visualized using the ggplot2 package.

### 2.7 Correlation analysis

Pearson’s correlation analysis between the expression of hub genes and the abundance of immune effector cells was carried out to further explore the immunomodulatory mechanisms.

### 2.8 Prediction of transcription factor and drug-hub gene interaction

TRRUST (https://www.grnpedia.org/trrust/) (Han et al., 2018), a database that provides transcription factor-target regulatory relationships, was applied to predict transcription factors that regulate hub gene expression. The expression levels of transcription factors predicted by TRRUST were confirmed in GSE69063 (anaphylaxis) and GSE60993 (STEMI), and the differences between diseased and healthy groups were compared using *t*-test. ChEA3 (ChIP-X Enrichment Analysis, version 3) platform provides transcription factor enrichment analysis and integrates RNA-seq data from GTEx, TCGA, and ARCHS4, as well as CHIP-seq data from ENCODE and ReMap (Keenan et al., 2019). The hub gene list was submitted to ChEA3, and transcription factors common to those predicted by TRRUST were identified. Base on the MeanRank method, transcription factors were ranked according to their composite scores. DGIdb (Drug-gene interaction database, http://www.dgidb.org) was utilized to predict the drug-hub gene interaction (Freshour et al., 2021).

### 2.9 Murine model

C57BL/6 mice were obtained from the Charles River Laboratories (Beijing, China). The murine model used in this study was based on an active systemic anaphylaxis model described previously (Jonsson et al., 2011) with modifications. Six-week-old female mice were sensitized subcutaneously on day 0 with bovine serum albumin (BSA, 50 μg per mouse, Sigma-Aldrich, United States) in complete Freund adjuvant (CFA, Sigma-Aldrich, United States) and boosted on day 7 and day 14 with 50 μg BSA in incomplete Freund adjuvant (IFA, Sigma-Aldrich, United States). One week after the last sensitization, mice were intravenously injected with 15 μg BSA to elicit systemic anaphylaxis. After BSA challenge, the temperature was monitored every 10 min with a rectal thermometer (TH212,China), and the severity of anaphylaxis was scored every 10 min on a scale of 0–4 based on the grading system described previously (Cloutier et al., 2018), score 0: normal; score 1: slow motions; score 2: impaired mobility, still reacting to touch; score 3: immobilized and do not react to touch; score 4: death.

### 2.10 Electrocardiogram recording

When fully anesthetized with inhalant isoflurane (RWD, China), mice were immobilized in a supine position with electrodes implanted subcutaneously in the limbs. The Animal Bio Amp device (ADInstruments, Australia) and LabChart software were used to acquire and record the lead II ECGs of naïve and model mice.

### 2.11 Heart function assessment

One day prior to ultrasound, hair around the chest wall was carefully removed using depilatory cream (Nair, United States). Mice were anesthetized by inhalant isoflurane (RWD, China), at a concentration of 1.5%. When fully anesthetized, the mouse was positioned on the warm imaging platform ventral side up, and the medical ultrasonic gel was applied to the limb leads to generate ECG. Vevo2100 System (Visual Sonics, Canada) was used to record the transthoracic echocardiograms of naïve and model mice. The MS550D transducer was placed on the left sternal edge to obtain a parasternal short axis (PSAX) view. Left ventricular ejection fraction (EF) and fractional shortening (FS) were acquired from PSAX M-mode scans at the mid-papillary muscle level. Echocardiographic data were analyzed offline using Vevo Lab software and all measurements were averaged from 3 cardiac cycles.

### 2.12 Quantitative real-time polymerase Chain reaction

Total RNA was extracted from the peripheral blood of naïve mice and model mice using TRIzol reagent (Invitrogen, United States). PrimeScript™ RT Master Mix (TaKaRa, Japan) was utilized to convert the equivalent amount (500 ng) of total RNA into cDNA. The relative mRNA expression of target genes was quantified with the SYBR master mix (Yeasen, China). All samples were normalized to the housekeeping gene *β-Actin*. Details of primers are listed as follows.

**Table:** 

Gene	Forward sequence (5′-3′)	Reverse sequence (5′-3′)
IL1R2	TCC​GGG​TCA​AAG​GAA​CAA​CC	CCC​AGA​AAC​ACT​TTG​CAC​GG
FOS	TAC​TAC​CAT​TCC​CCA​GCC​GA	GCT​GTC​ACC​GTG​GGG​ATA​AA
MMP9	TAG​ATC​ATT​CCA​GCG​TGC​CG	GCC​TTG​GGT​CAG​GCT​TAG​AG
DUSP1	ATCGTGCCCAACGCTGAA	GAA​AAC​GCT​TCA​TAT​CCT​CCT​TGG
CLEC4D	ACT​GAT​CCC​TTG​CGT​CTT​CG	CGG​ATG​CAC​GTT​ACT​CTC​GT
CREM	TGG​AAA​CAG​TTG​AAT​CAC​AGC​A	ATC​TTG​GGA​ATA​CCA​GGC​ACA
SRF	GGC​CGC​GTG​AAG​ATC​AAG​AT	CAC​ATG​GCC​TGT​CTC​ACT​GG
STAT6	CTC​TGT​GGG​GCC​TAA​TTT​CCA	CAT​CTG​AAC​CGA​CCA​GGA​ACT
STAT3	CAA​TAC​CAT​TGA​CCT​GCC​GAT	GAG​CGA​CTC​AAA​CTG​CCC​T
SP1	GCC​GCC​TTT​TCT​CAG​ACT​C	TTG​GGT​GAC​TCA​ATT​CTG​CTG
β-Actin	TTA​CTG​CTC​TGG​CTC​CTA​GC	CAG​CTC​AGT​AAC​AGT​CCG​C

### 2.13 Logistic regression analysis

SPSS version 23.0 (SPSS Inc., Chicago, IL, United States) was used to perform univariate logistic analysis and construct multivariate logistic regression model with the expression profiles of hub genes as the continuous prediction variable and the physical condition (disease or not) as the categorical response variable. Statistically significant variables (*p* < 0.05) were included in the multivariate logistic regression model using the backward LR method for variable selection. ROC curves were plotted and the AUC value was calculated to evaluate the diagnostic efficiency of hub genes.

### 2.14 Statistics.

Statistical analysis was performed using R language (R Foundation for Statistical Computing, Vienna, Austria) and GraphPad Prism 8.0 software (GraphPad Software, San Diego, CA, United States). Data were evaluated for normal distribution using Shapiro-Wilk tests. If data were normally distributed and with similar variances, two-tailed Student’s t-test (parametric) was used to compare the differences between two groups. For normally distributed data with unequal variances, unpaired *t*-test with Welch’s correction was performed. For abnormally distributed data with unequal variances, Kolmogorov-Smirnov test (nonparametric) was used to determine differences between two groups. Pearson’s correlation test was used to analyze the correlation between the expression of hub genes and the abundance of immune effector cells. Quantitative data are shown as mean ± SEM. *p < 0.05, **p < 0.01, ***p < 0.001 were considered statistically significant.

## 3 Results

### 3.1 Transcriptomic signatures of acute anaphylaxis

**FIGURE 1:**
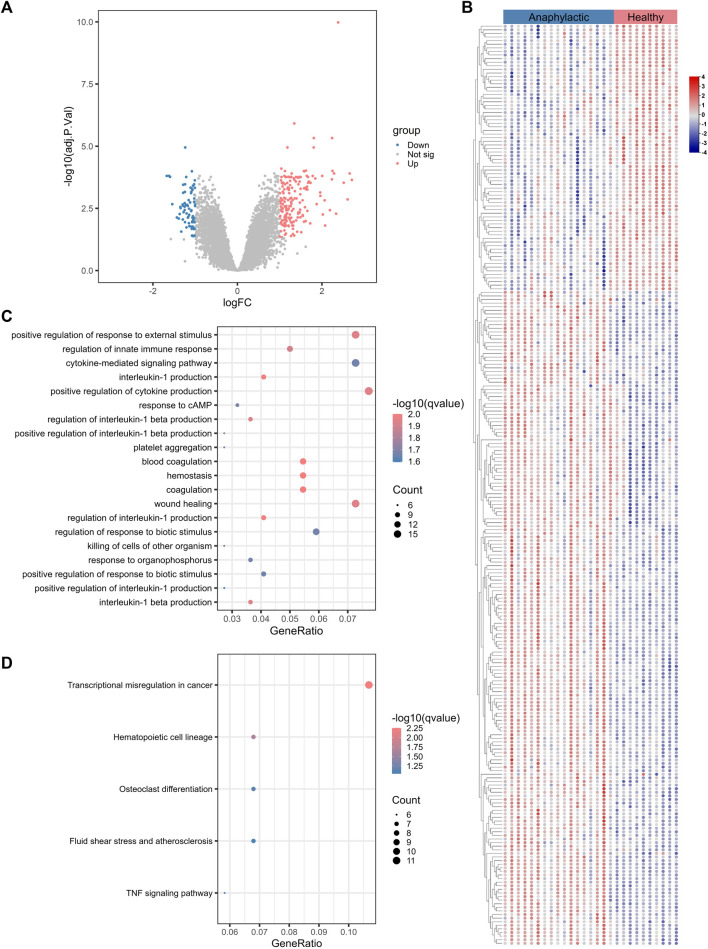
Transcriptomic signature of acute anaphylaxis. **(A)**, Volcano plot of transcripts from GSE69063 (anaphylaxis dataset), where blue indicates downregulated DEGs, red represents upregulated DEGs, and gray indicates genes without significant differences. **(B),** Heatmap of DEGs from GSE69063. Blue indicates downregulated DEGs and red represents upregulated DEGs. **(C)** GO analysis of DEGs from GSE69063. The color indicates the -log10(qvalue) of the biological process (BP) terms, and the count represents the number of genes enriched in a BP term. **(D)**, KEGG analysis of DEGs.

The workflow of this study was shown in Supplementary Figure S1. To characterize the gene expression profile of acute anaphylaxis, we compared mRNA expression in whole blood between anaphylactic patients and healthy subjects. Venous blood was collected at 1 hour after patients’ arriving at the emergency department. A total of 265 genes were differentially expressed in the anaphylactic patients, among which 188 were upregulated and 77 were downregulated (Figure 1A). The heatmap revealed high heterogeneity of gene expression between anaphylactic patients and healthy controls (Figure 1B). To explore the pathogenesis of anaphylaxis, we performed GO and KEGG analysis, and profiling of biological processes and signaling pathways revealed the complex pathology of anaphylaxis. This disease was featured by marked activation of immune-inflammatory responses, including positive regulation of response to external stimulus, regulation of innate immune response, cytokine-mediated signaling pathways, and positive regulation of cytokine production, especially interleukin-1 (Figure 1C). In addition, the blood coagulation cascade, platelet aggregation, hemostasis, and wound healing actively participated in the development of anaphylaxis (Figure 1C). KEGG results illustrated that anaphylaxis-related DEGs were associated with transcriptional misregulation and hematopoietic cell lineage (Figure 1D).

### 3.2 Leukocyte composition of acute anaphylaxis

**FIGURE 2:**
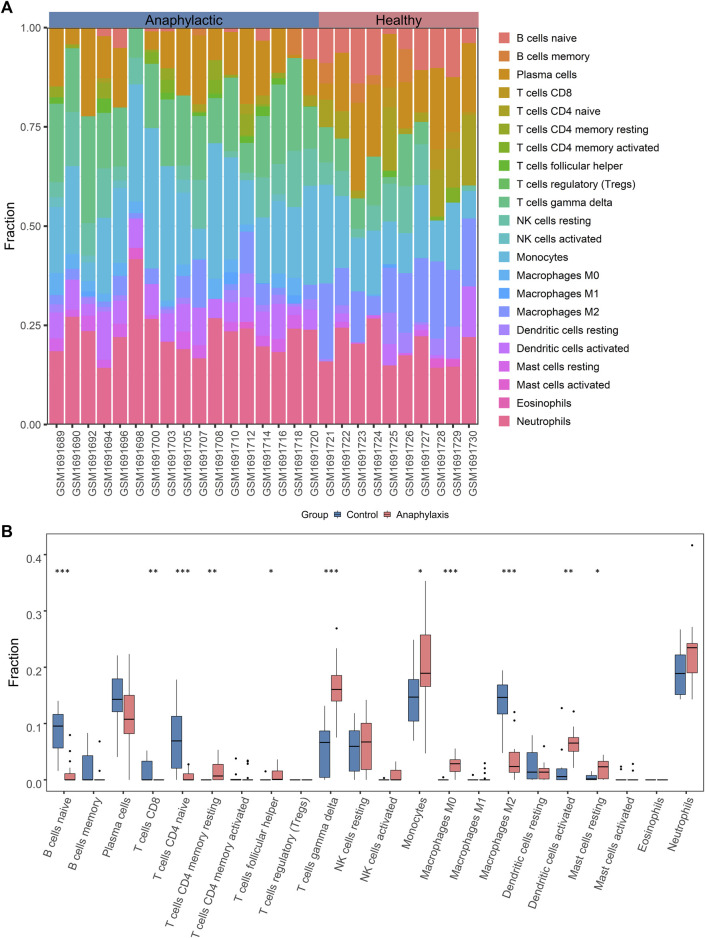
Immune cell characterization of acute anaphylaxis. (**A)**, Histogram of the distribution of 22 immune cell subtypes in GSE69063 (anaphylaxis dataset). **(B)**, Boxplot of the fraction of 22 immune cell subtypes in control and anaphylactic specimens.

Since anaphylaxis was characterized by marked activation of immune-inflammatory responses, the CIBERSORT deconvolution algorithm was applied to further explore the details of immune cells involved in acute anaphylaxis. As shown in Figure 2A, a wide heterogeneity of immune cell distribution was observed in anaphylactic patients compared to healthy subjects. Among the 22 immune cell subtypes, CD4^+^ memory T cells (resting), follicular helper T cells, and M0 macrophages were distributed only in anaphylactic samples but not in controls. Compared to the controls, anaphylactic specimens showed significantly increased levels of T cells gamma delta, monocytes, dendritic cells (activated) and mast cells (resting) and markedly decreased proportions of naïve B cells, CD8^+^ T cells, CD4^+^ naïve T cells, and M2 macrophages (Figure 2B).

### 3.3 Transcriptomic signatures of STEMI

**FIGURE 3:**
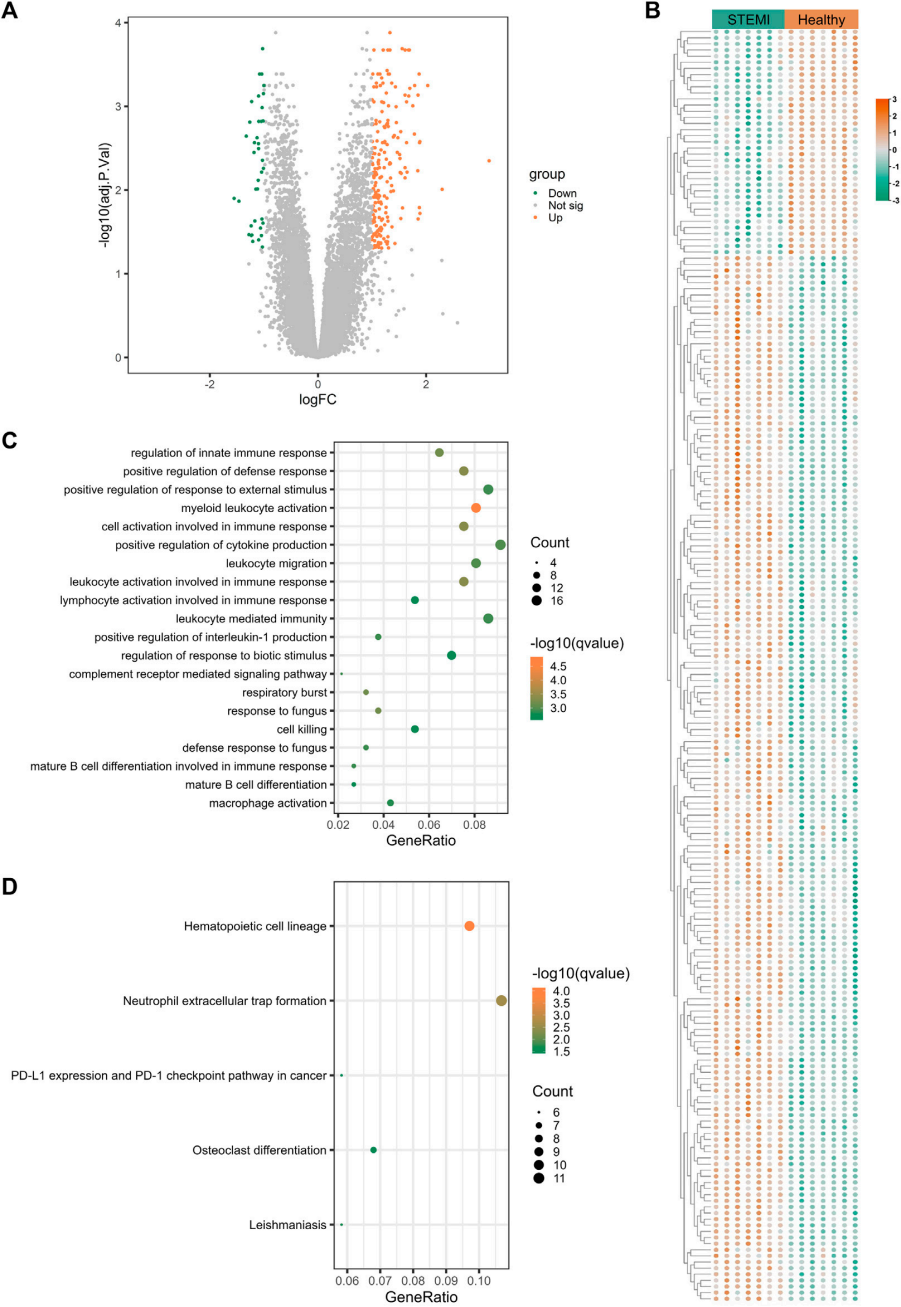
Transcriptomic signature of STEMI. (**A)**, Volcano plot of transcripts from GSE60993 (STEMI dataset), where green indicates downregulated DEGs, orange represents upregulated DEGs, and gray indicates genes without significant differences. **(B)**, Heatmap of DEGs from GSE60993. Green indicates downregulated DEGs and orange represents upregulated DEGs. **(C)** GO enrichment analysis of DEGs from GSE60993. The color indicates -log10(qvalue) of the BP terms, and the count represents the number of genes enriched in a BP term. (**D)**, KEGG analysis of DEGs from GSE60993.

To uncover the gene expression profiles of STEMI, we compared the mRNA expression in whole blood between STEMI patients and healthy subjects. A total of 237 STEMI-related DEGs were screened, including 199 upregulated and 38 downregulated genes, and these DEGs were visualized with the volcano plot and heatmap (Figures 3A,B). STEMI-related DEGs were primarily involved in the biological processes of innate immune response regulation, positive regulation of defense response, and positive regulation of response to external stimulus, indicating immune defense responses were essential in STEMI. Notably, myeloid leukocyte activation, positive regulation of cytokine production, leukocyte migration, and leukocyte-mediated immunity were critically involved in the development of STEMI (Figure 3C). KEGG analysis revealed that STEMI-related DEGs were associated with hematopoietic cell lineage, neutrophil extracellular trap formation, PD-L1 expression and PD-1 checkpoint pathway (Figure 3D).

### 3.4 Leukocyte composition of STEMI

**FIGURE 4:**
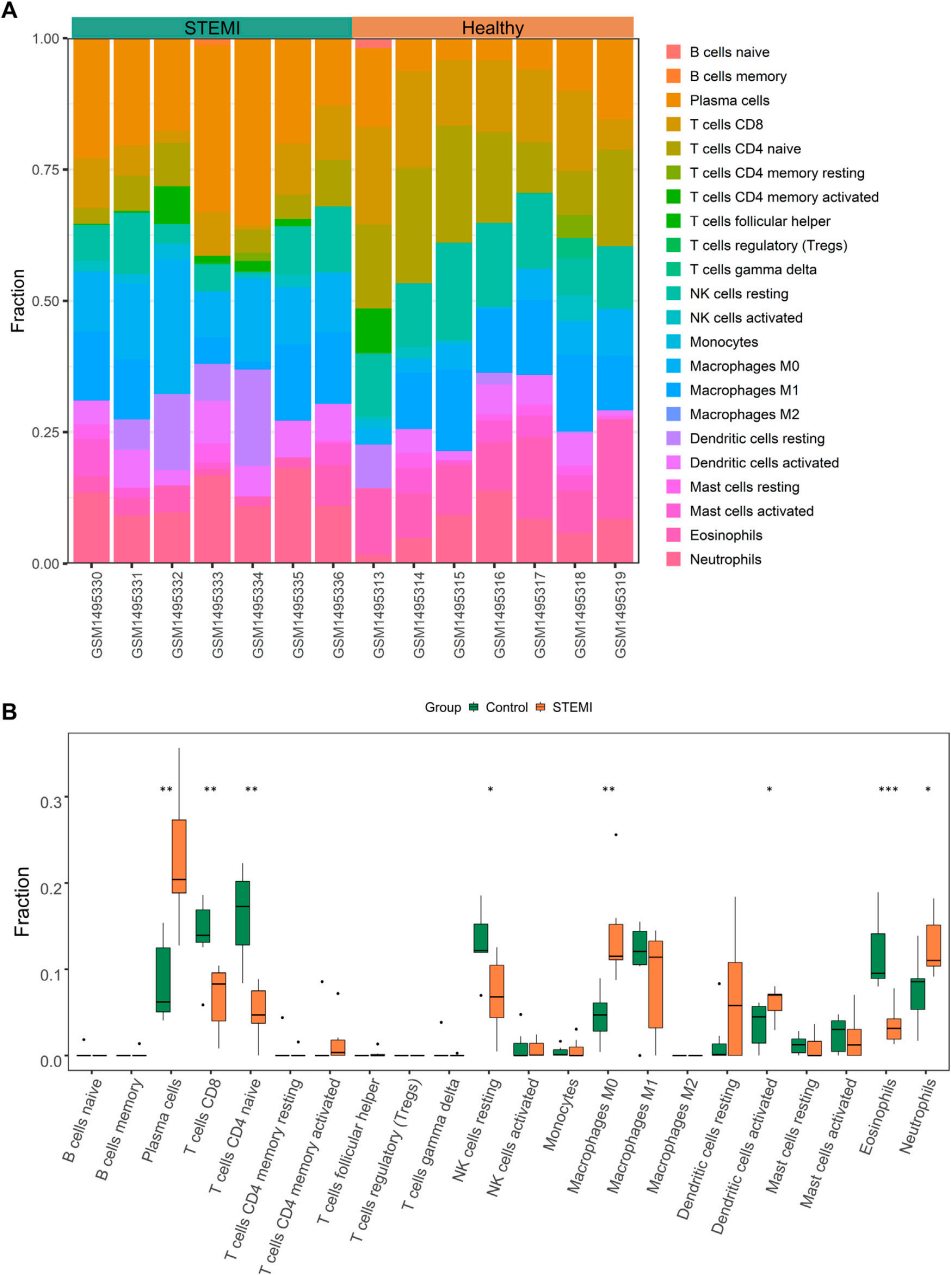
Immune cell characterization of STEMI. **(A)**, Histogram of the composition and distribution of 22 immune cell subtypes in GSE60993 (STEMI dataset). **(B)**, Boxplot of the fraction of 22 immune cell subtypes in control and STEMI specimens.

As highlighted above, myeloid leukocyte activation and leukocyte-mediated immunity were essential in STEMI. To further elucidate components of immune cells key to STEMI pathology, we used CIBERSORTx to profile the composition and distribution of immune cell subtypes in STEMI and normal samples. As shown in Figure 4A, there were marked differences in the distribution of immune cells between these two groups. STEMI samples were featured by increased levels of plasma cells, M0 macrophages, dendritic cells (activated), and neutrophils, and by decreased infiltration of CD8^+^ T cells, CD4^+^ naïve T cells, NK cells (resting), and eosinophils (Figure 4B).

### 3.5 Identification of hub genes shared by anaphylaxis and STEMI

**FIGURE 5:**
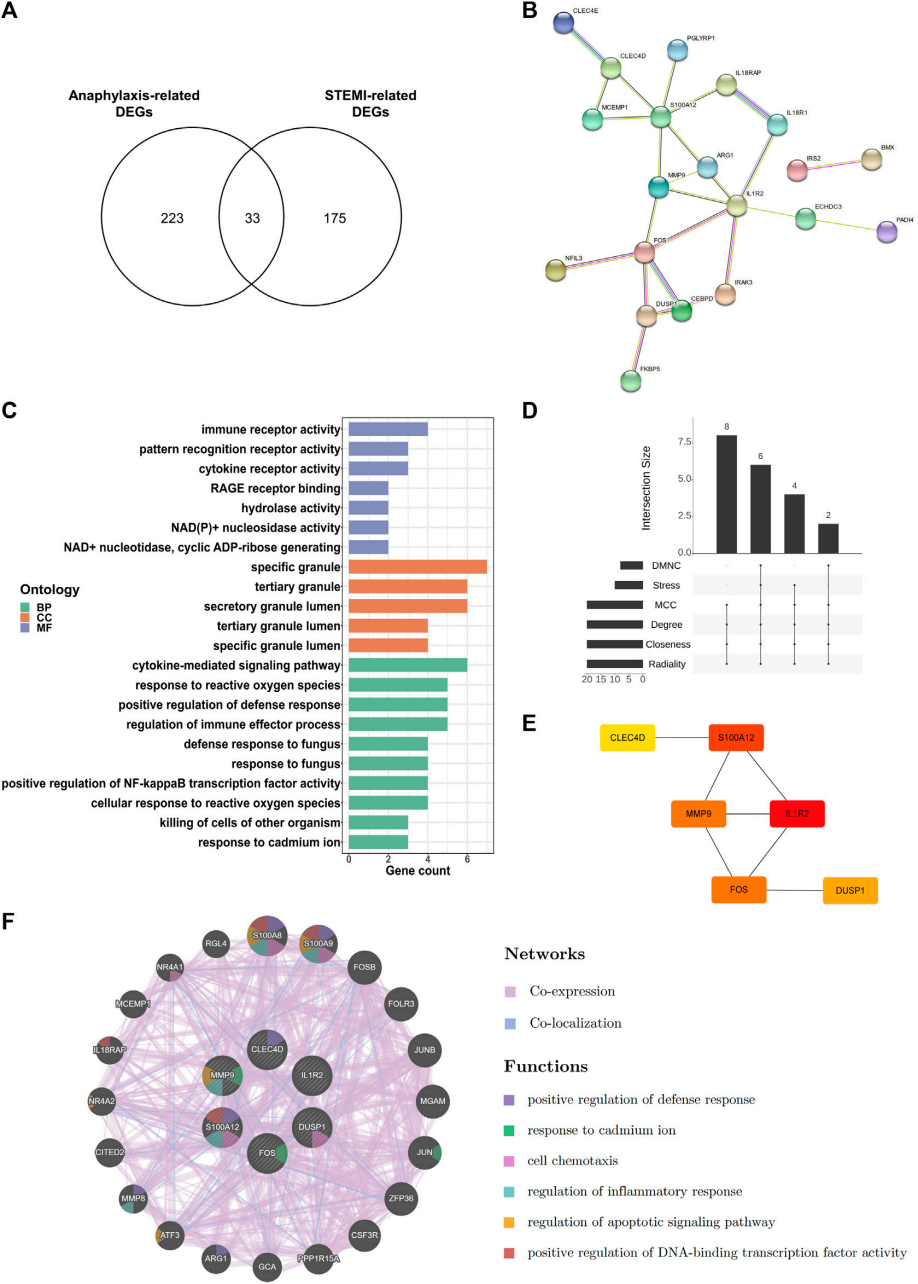
Key transcripts shared by anaphylaxis and STEMI. **(A)**, Venn diagram of anaphylaxis-related DEGs and STEMI-related DEGs. **(B)**, The PPI network of 33 common DEGs. **(C)**, GO analysis of 33 common DEGs from three perspectives: biological process (BP), cellular component (CC), and molecular function (MF). **(D)**, The Venn diagram showing six hub genes at the intersection of six algorithms (DMNC, Stress, MCC, Degree, Closeness, Radiality). **(E)**, Interactions of six hub genes exhibited by cytoHubba. **(F)**, Co-expression and co-localization network of hub genes constructed by GeneMANIA.

By extracting DEGs that were shared by anaphylaxis and STEMI, 33 common DEGs were identified (Figure 5A). The PPI network of these common DEGs contained 33 nodes and 24 edges, revealing complex interactions among these molecules (Figure 5B). These common DEGs were enriched in the secretory granules, and their molecular functions include immune receptor activity, pattern recognition receptor activity, and cytokine receptor activity (Figure 5C), suggesting immune-inflammatory regulation is essential in both anaphylaxis and STEMI. Enrichment analysis of these common DEGs demonstrated that the shared biological processes of anaphylaxis and STEMI included cytokine-mediated signaling pathway, response to reactive oxygen species, positive regulation of defense response, regulation of immune effector process, and positive regulation of NF-kappa B transcription factor activity (Figure 5C). Taking the intersection of six algorithms (DMNC, Stress, MCC, Degree, Closeness, Radiality) in cytoHubba (Figure 5D), we found 6 hub genes shared by anaphylaxis and STEMI: IL1R2, S100A12, FOS, MMP9, DUSP1, and CLEC4D (Figure 5E). The description and major functions of hub genes are listed in Supplementary Table S2. These hub genes constituted an interactive network with a co-expression rate of 96.23% and a co-localization rate of 3.77% (Figure 5F). The prominent functions of hub genes included positive regulation of defense response, response to cadmium ion, cell chemotaxis, regulation of inflammatory response, regulation of apoptotic signaling pathway, and positive regulation of DNA-binding transcription factor activity (Figure 5F).

### 3.6 Correlation analysis between hub genes and immune cells

**FIGURE 6:**
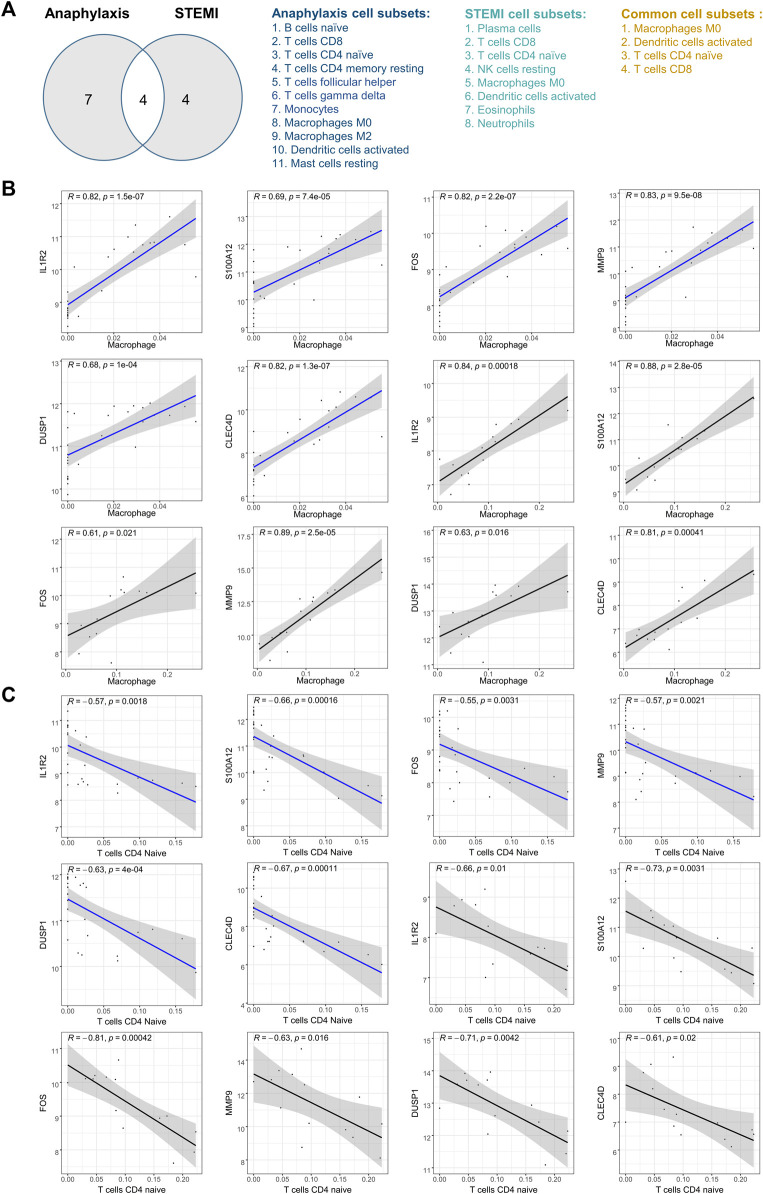
Pearson’s correlation analysis between hub genes and immune effector cells. **(A)**, Venn diagram of anaphylaxis-related cell subsets and STEMI-related cell subsets. **(B)**, Scatter diagrams of the correlations between hub gene expression and M0 macrophage abundance in anaphylactic samples (blue) and STEMI samples (black). (**C)**, Scatter diagrams of the correlations between hub gene expression and CD4^+^ naïve T cell abundance in anaphylactic samples (blue) and STEMI samples (black). R > 0 indicates positively correlated and R < 0 indicates negatively correlated. *p* < 0.05 was considered statistically significant.

After taking the intersection and excluding the immune cells with opposite infiltration trends in anaphylactic and STEMI groups, M0 macrophages, activated dendritic cells, CD4^+^ naïve T cells, and CD8^+^ T cells were identified as immune effector cells associated with both anaphylaxis and STEMI (Figure 6A). To further investigate the association of hub genes and these immune cell subtypes, Pearson’s correlation analysis was performed on the expression levels of hub genes and the abundance of immune cells in both anaphylactic and STEMI samples. The results illustrated that all hub genes were positively correlated with M0 macrophage (R > 0, *p* < 0.05), while negatively correlated (R < 0, *p* < 0.05) with CD4^+^ naïve T cells (Figures 6B,C **)**. FOS showed a significant positive correlation with activated dendritic cells, and CLEC4D had a significant negative correlation with CD8^+^ T cells (Supplementary Figure S2A, B).

### 3.7 Verification of hub gene expression in external cohorts

**FIGURE 7:**
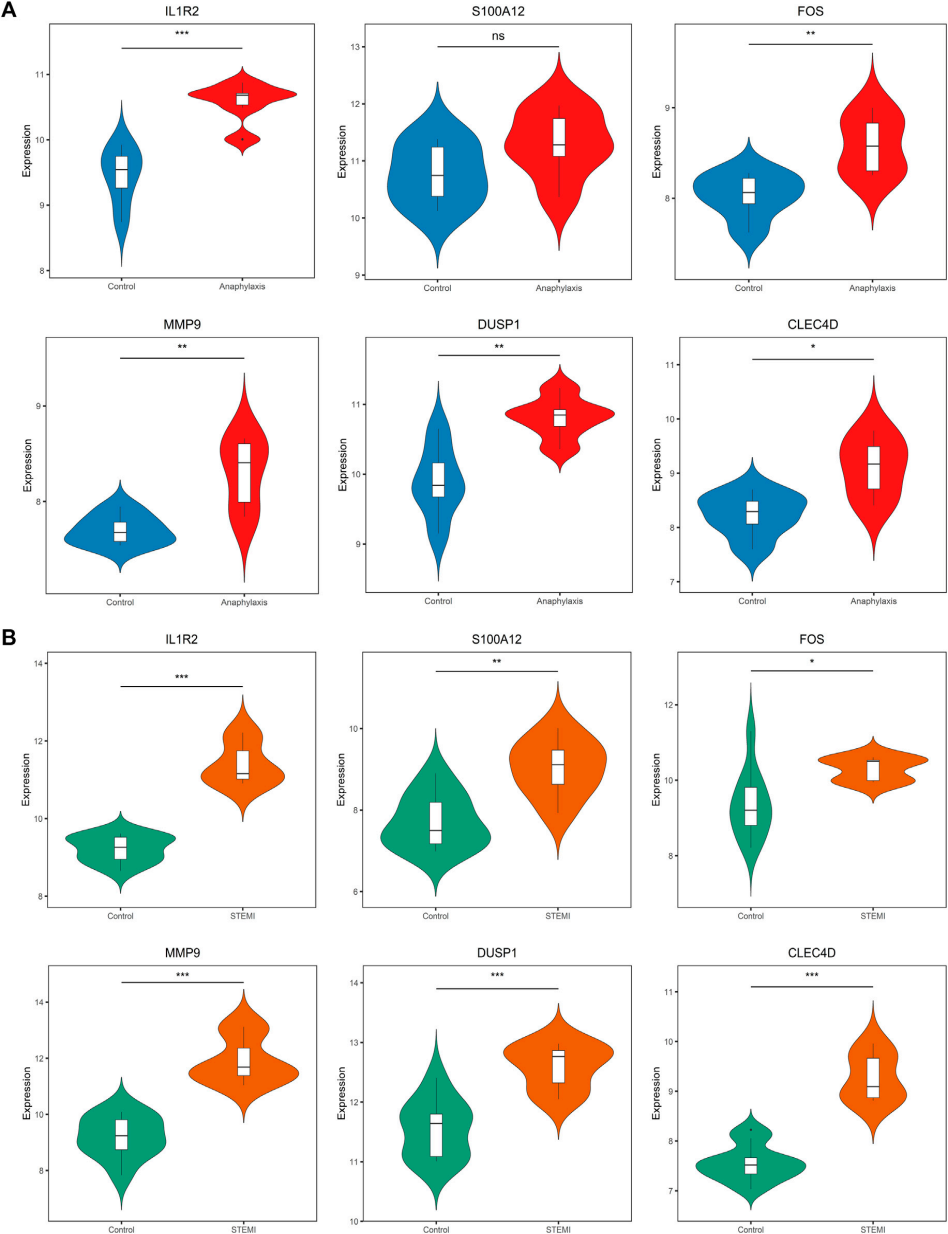
Validation of hub gene expression in GSE47655 (anaphylaxis) and GSE61144 (STEMI) datasets. (**A)**, Validation of hub gene expression (IL1R2, S100A12, FOS, MMP9, DUSP1, CLEC4D) in GSE47655 (anaphylaxis dataset). (**B)**, Validation of hub gene expression (IL1R2, S100A12, FOS, MMP9, DUSP1, CLEC4D) in GSE61144 (STEMI dataset).

To assess the robustness of hub genes identified above, we analyzed their gene expression in external human microarray datasets (GSE47655 for anaphylaxis and GSE61144 for STEMI) (Supplementary Table S1). Compared with healthy controls, 5 hub genes (IL1R2, FOS, MMP9, DUSP1, CLEC4D) were significantly upregulated in anaphylactic samples, while S100A12 showed no statistical difference (Figure 7A). In STEMI samples, the expression of all hub genes (IL1R2, S100A12, FOS, MMP9, DUSP1, CLEC4D) was markedly higher than that of controls (Figure 7B).

### 3.8 Validation of hub gene expression in a murine model of acute anaphylaxis

**FIGURE 8:**
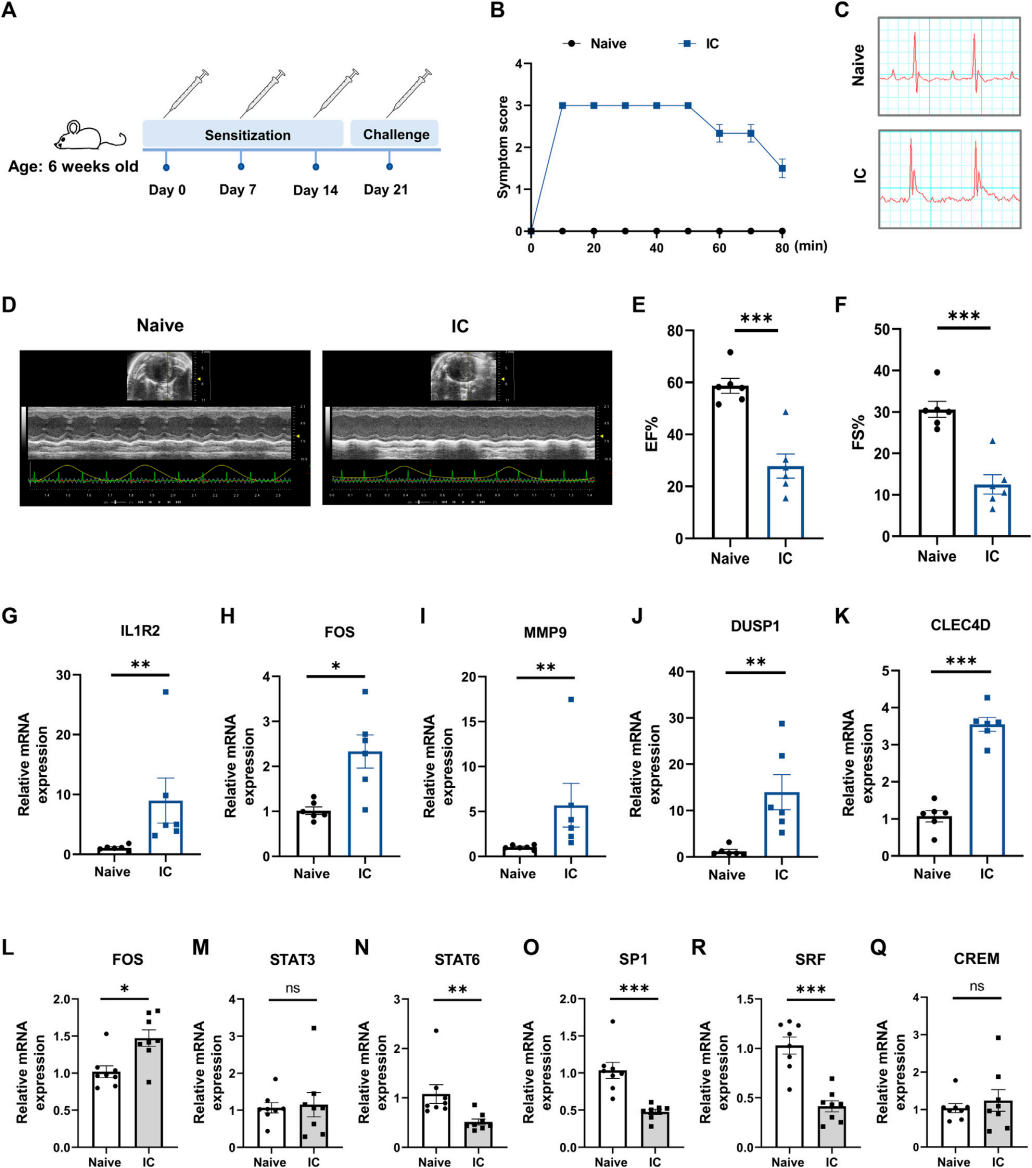
Verification of hub gene expression in a murine model of anaphylaxis complicated with STEMI. (**A)**, Schematic diagram of the experimental protocol for establishing the murine model. 6-week-old female mice were subcutaneously sensitized on day 0 with 50 μg BSA in CFA and boosted subcutaneously on day 7 and day 14 with 50 μg BSA in IFA. On day 21, mice were intravenously injected with 15 μg BSA to induce systemic anaphylaxis. After the BSA challenge, the anaphylactic mice showed acute and transient ST-segment elevations on electrocardiograms. (**B)**, Severity of anaphylactic response was scored on a scale of 0–4 (n = 6 per group). Score 0: normal; score 1: slow motions; score 2: impaired mobility, still reacting to touch; score 3: immobilized and does not react to touch; score 4: death. (**C)**, Representative electrocardiograms of naïve mice and model mice. (**D)** through **(F)**, Heart function was measured with echocardiography 10 min after BSA challenge. Representative echocardiogram (**D**), ejection fraction (EF%) (**E**), and fractional shortening (FS%) (**F**) of naïve mice and model mice (n = 6 per group). **(G)** through (**K)**, qRT-PCR analysis of IL1R2 (**G**), FOS (**H**), MMP9 (**I**), DUSP1 (**J**), and CLEC4D (**K**) in peripheral whole blood (n = 6 per group). (**L**) through (**Q**), qRT-PCR analysis of FOS (**L**), STAT3 (**M**), STAT6 (**N**), SP1 (**O**), SRF (**P**), and CREM (**Q**) in peripheral whole blood (n = 8 per group). Naïve indicates untreated control mice. IC indicates BSA-treated model mice. *p < 0.05, **p < 0.01, ***p < 0.001. Statistical analysis: unpaired Student’s t-test (**E**, **F**, **K**, **P**), Kolmogorov-Smirnov test (**G**, **I**, **J**, **N**, **O**), Unpaired *t*-test with Welch’s correction (**H**), Mann Whitney test (**L**).

To further verify the findings from human microarray data, the expression levels of five hub genes (IL1R2, FOS, MMP9, DUSP1, CLEC4D) were measured in an experimentally induced murine model of acute anaphylaxis by qRT-PCR analysis. Systemic anaphylaxis was induced by intravenously injected with BSA in mice sensitized with the same antigen (Figure 8A). 10 min after the BSA challenge, mice showed obvious symptoms of anaphylaxis, including impaired mobility, lethargy, and unresponsiveness (Figure 8B). Compared with naïve mice, the anaphylactic mice exhibited ST-segment elevations on electrocardiograms (Figure 8C) and showed markedly impaired heart function (Figures 8D–F) as reflected by decreases in ejection fraction (EF; 58.68% ± 2.87% *versus* 27.84% ± 4.67%) and fractional shortening (FS; 30.62% ± 1.95% *versus* 12.53% ± 2.33%). qRT-PCR analysis of peripheral blood extracts demonstrated that the levels of IL1R2, FOS, MMP9, DUSP1, and CLEC4D in the anaphylactic mice with STEMI were significantly upregulated (Figures 8G–K), which was consistent with the human microarray results. Taken together, these results indicated that the hub genes we screened were of high reliability.

### 3.9 Prediction of transcription factor and candidate druggable hub genes

TRRUST database was applied to explore key upstream regulators for hub genes. Signal transducer and activator of transcription 3 (STAT3), cAMP-responsive element modulator (CREM), signal transducer and activator of transcription 6 (STAT6), Sp1 transcription factor (SP1), Fos proto-oncogene (FOS), serum response factor (SRF), and histone deacetylase 1 (HDAC1) were identified as transcription factors that modulated hub gene expression (Supplementary Figure S3A). The details of these transcription factors are summarized in Supplementary Table S3. With further analysis, we found that CREM and FOS were highly expressed in anaphylactic samples, while 3 transcription factors (SRF, STAT6, SP1) were significantly downregulated (Supplementary Figures S3B–H). In STEMI samples, 3 transcription factors (FOS, STAT3, SP1) were markedly upregulated, while HDAC1 was significantly downregulated (Supplementary Figures S3I–O). Moreover, ChEA3 platform was used to validate the findings from TRRUST and further rank the transcription factors according to their MeanRank composite scores. STAT3, CREM, STAT6, SP1, FOS, and SRF were identified as transcription factors of hub genes that common to those predicted by TRRUST, ranking as: FOS, STAT3, STAT6, SP1, SRF, and CREM (Supplementary Table S4). qRT-PCR analysis of peripheral blood extracts showed significantly increased expression of FOS, and markedly decreased expression of STAT6, SP1, and SRF in the murine model of anaphylaxis complicated STEMI (Figures 8L–Q), suggesting the important regulatory role of these transcription factors in anaphylaxis induced myocardial damage.

The candidate druggable hub genes were predicted via the DGIdb database. As listed in Table 1, IL1R2, FOS, MMP9, and DUSP1 showed interactions with approved drugs, suggesting the potential targeting and regulatory effects of approved drugs on these hub genes.

**TABLE 1:** Potential drug-hub gene interaction predicted by DGIdb database.

Gene	Potential drug	Interaction score	Drug class	Drug indication
IL1R2	ANAKINRA	7.73	IL1R antagonist	Rheumatoid arthritis
FOS	BACLOFEN	1.24	Muscle relaxant	Multiple sclerosis
MMP9	ANDECALIXIMAB	10.30	MMP9 antibody	Gastric cancer
DUSP1	ALBUTEROL	6.87	β2 adrenergic agonist	Acute asthma
CLEC4D	N/A	N/A	N/A	N/A

**Abbreviations:** DGIdb, drug-gene interaction database; IL1R, interleukin-1, receptor; CGRP, calcitonin gene-related peptide; FOS, Fos proto-oncogene; MMP9, matrix metallopeptidase 9; DUSP1, dual specificity phosphatase 1; CLEC4D, C-type lectin domain family 4 member D.

### 3.10 Identification of diagnostic efficiency of hub genes on anaphylaxis and STEMI

**FIGURE 9:**
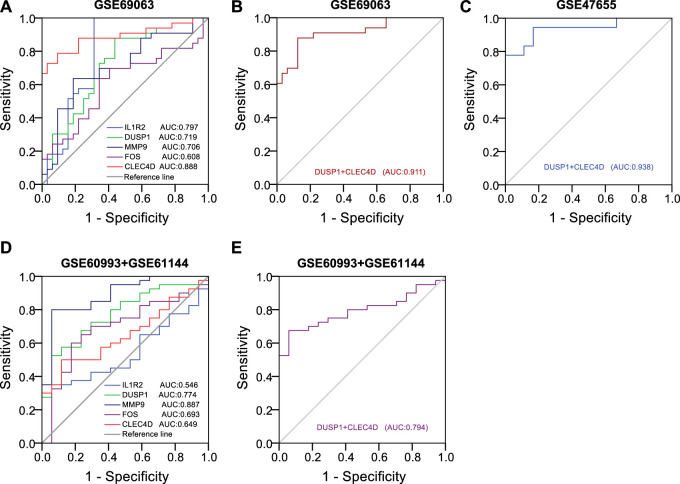
Identification of diagnostic efficiency of hub genes based on logistic regression models. (**A)**, Receiver operating characteristic (ROC) curves of five hub genes in the anaphylactic dataset (GSE69063). (**B)**, ROC curves of DUSP1+CLEC4D in the anaphylactic dataset (GSE69063). (**C)**, Validation of the diagnostic efficiency of DUSP1+CLEC4D in the independent anaphylactic dataset (GSE47655). (**D)**, ROC curves of five hub genes in the STEMI datasets (GSE60993 and GSE61144). (**E)**, Validation of the diagnostic efficiency of DUSP1+CLEC4D in the STEMI datasets (GSE60993 and GSE61144).

To predict the occurrence of anaphylaxis and STEMI, univariate logistic analysis followed by multivariate logistic regression analysis was applied to construct the diagnostic model, and receiver operating characteristic (ROC) curves were used to evaluate the diagnostic efficiency of hub genes. Univariate logistic analysis of hub genes in anaphylactic dataset GSE69063 revealed that IL1R2 (*p* < 0.001, AUC = 0.797, 95% CI 0.679-0.916), DUSP1 (*p* = 0.002, AUC = 0.719, 95% CI 0.593-0.844), MMP9 (*p* = 0.003, AUC = 0.706, 95% CI 0.577-0.835), and CLEC4D (*p* < 0.001, AUC = 0.888, 95% CI 0.803-0.974) were significantly correlated with anaphylaxis as continuous variables (Figure 9A). All statistically significant variables were then included in the multivariate logistic regression model using the backward LR method for variable selection. The results showed DUSP1 (*p* = 0.028) and CLEC4D (*p* = 0.001) were selected as significant variables and used to construct diagnostic model. The regression equation of logit (P) = −33.355 + 2.9*DUSP1+2.294*CLEC4D was established and the accuracy of this logistic model was evaluated by ROC curves. AUC values in GSE69063 and another independent anaphylactic dataset GSE47655 were 0.911 (95% CI, 0.840-0.982) and 0.938 (95% CI, 0.857-1.000), respectively (Figures 9B,C), suggesting this model was of high diagnostic efficacy in distinguishing anaphylactic patients from healthy individuals. As anaphylaxis and STEMI shared common hub genes, the diagnostic efficiency of hub genes was further evaluated in STEMI datasets. Univariate logistic analysis of hub genes in STEMI dataset GSE60993 and GSE61144 revealed that DUSP1 (*p* = 0.001, AUC = 0.774, 95% CI 0.649-0.898), MMP9 (*p* < 0.001, AUC = 0.887, 95% CI 0.794-0.980), and CLEC4D (*p* = 0.03, AUC = 0.649, 95% CI 0.507-0.790) were significantly correlated with STEMI (Figure 9D). Next, the efficiency of the established diagnostic model was validated in the STEMI datasets (GSE60993 and GSE61144) based on the expression profiles of DUSP1 and CLEC4D. As shown in Figure 9E, the AUC values in STEMI datasets exceeded 0.7, indicating this model also had good accuracy in discriminating STEMI. Taken together, these results suggested that two hub genes, DUSP1 and CLEC4D, held a promise for the diagnosis of anaphylaxis complicated STEMI as blood diagnostic biomarkers.

## 4 Discussion

STEMI following anaphylaxis is an underdiagnosed but potentially fatal disease (Roumeliotis et al., 2021). The pathogenesis of anaphylaxis complicated STEMI remains poorly understood and the early diagnosis and treatment have not been fully established. The present study demonstrates the pivotal role of inflammation and defense responses in anaphylaxis and STEMI, uncovers five hub genes (IL1R2, FOS, MMP9, DUSP1, CLEC4D) closely correlated with immune effector cells of anaphylaxis and STEMI, and identifies two effective diagnostic markers (DUSP1and CLEC4D), which deepens our understanding of the pathogenesis of anaphylaxis complicated with STEMI and provides potential blood diagnostic biomarkers and therapeutic targets for this complication.

In anaphylactic patients with pre-existing atherosclerosis, STEMI is associated with allergic factors-triggered atheromatous plaque erosion or stent thrombosis, which culminates in coronary occlusion and myocardial damage (Sakaue et al., 2020; Engheta et al., 2021; Yamamoto et al., 2022). While in patients with angiographically normal coronary arteries and no risk factors for coronary artery disease, prolonged coronary spasm appears to be the leading cause of acute STEMI following anaphylaxis (Goto et al., 2016; Li et al., 2018). Mast cells, distributed around coronary arteries and plaques, have been proposed to be implicated in anaphylaxis complicated with STEMI (Marone et al., 2014; Li et al., 2018; Yamamoto et al., 2022). Upon activation, mast cells secrete inflammatory mediators such as histamine (Galli and Tsai, 2012; Galli et al., 2020), which regulates coronary artery tone and vascular permeability, thus affecting hemodynamic stress and coronary blood flow (Matsuyama et al., 1990; Kounis and Zavras, 1991; Mikelis et al., 2015). In this study, we observed specific immune cell subsets, including macrophages, dendritic cells, CD8^+^ T cells, and CD4^+^ naïve T cells, which act in both anaphylaxis and STEMI (Figure 6A).

Macrophages express FcγR, and IgG/FcγR/macrophage pathway is implicated in the initiation of the immune-inflammatory cascade upon IgG-allergen immune complex stimulation (Beutier et al., 2017). Depletion of macrophages significantly attenuated both IgG2a- and IgG2b-mediated passive systemic anaphylaxis in mice (Beutier et al., 2017). During anaphylaxis, crosslinking of macrophage FcγR activates macrophages to release platelet-activating factor, an endogenous phospholipid mediator that may contribute to anaphylaxis-related STEMI by inducing platelet aggregation, endothelial dysfunction, inflammatory cell adhesion, and vascular hyperpermeability (Gill et al., 2015). Dendritic cells play essential roles in allergy sensitization, CD4^+^ naïve T cell activation, and T cell differentiation (Ruiter and Shreffler, 2012). Following uptake of exogenous allergens, dendritic cells integrate signals derived from exogenous allergens and present processed allergen to CD4^+^ naïve T cells through the peptide-MHC II-TCR and co-stimulatory signaling, leading to allergen-specific activation and expansion of CD4^+^ naïve T cells. Communication between dendritic cells and CD4^+^ T cells during allergen presentation further elicits Th2-type allergic responses, triggering the release of type 2 cytokines, IgE production, and accumulation of mast cells (Ruiter and Shreffler, 2012; Walker and McKenzie, 2018). Once re-exposure to allergen, allergen-IgE complex induced crosslinking of FcɛR receptors rapidly triggers mast cell activation and secretion of allergic chemicals (e.g., histamine and tryptase) that initiate immediate clinical symptoms (Galli and Tsai, 2012; Galli et al., 2020). Taken together, the above results and reported studies strongly suggest that anaphylaxis is a systemic inflammatory disease with extensive involvement of immune cells (increases in macrophage, activation of dendritic cells, differentiation of T cells, degranulation of mast cells). The episodes of STEMI secondary to anaphylaxis might be elicited by inflammatory factors-mediated direct stimulation to coronary intima or indirect hemodynamic change-triggered myocardial injury.

IL1R2, FOS, MMP9, DUSP1, and CLEC4D were screened and identified as hub genes shared by anaphylaxis and STEMI (Figures 5D,E). IL1R2 is expressed by monocytes/macrophages, T cells, and other immune cells (Boraschi et al., 2018). It has been reported that plasma levels of IL1R2 are profoundly increased in patients with STEMI and IL1R2 levels correlate independently with the adverse remodeling of left ventricle after STEMI, indicating the pivotal role of IL1R2 in myocardial injury and infarct healing (Orrem et al., 2018). Moreover, IL1R2 showed significantly increased expression in the whole blood of peanut-allergic subjects (Watson et al., 2017). FOS has been implicated in the regulation of degranulation capacity and inflammatory responses in FcεRI-activated mast cells (Lee et al., 2004). Inhibition of FOS expression by T-5224 attenuates IgE-mediated anaphylaxis (Wang et al., 2021). MMP9 is mainly involved in leukocyte migration and proteolysis. The levels of MMP9 are markedly increased in post-myocardial infarction patients, and high MMP9 is an independent predictor of 2-year adverse cardiovascular events (Webb et al., 2006; Kelly et al., 2008; Somuncu et al., 2020). DUSP1 regulates the MAPK signaling pathway via dephosphorylating threonine and tyrosine (Liu et al., 2007). Jana V. Maier et al. demonstrated that DUSP1-deficient mice showed high susceptibility to passive anaphylaxis (Maier et al., 2007). CLEC4D is a calcium-dependent pattern-recognition receptor that interacts with FcγR and forms a receptor complex. Binding of pathogens to this complex induces phosphorylation of ITAM, which facilitates activation of CARD9 and NF kappa B, consequently triggering antigen-presenting cell maturation and T cell differentiation (Miyake et al., 2013; Zhu et al., 2013). As shown in Figure 5F, these hub genes were primarily involved in the regulation of defense response, cell chemotaxis, and inflammatory response and shared a complex co-expression and co-localization network. Besides, these hub genes were closely correlated with macrophages and CD4^+^ naïve T cells (Figure 6), and the crosstalk between hub genes and special immune effector cell subsets potentially regulates the ongoing immune-inflammatory responses during anaphylaxis and STEMI.

Based on TRRUST and ChEA3 database, FOS, STAT3, STAT6, SP1, SRF, and CREM were identified as the upstream regulators for hub genes. Further qRT-PCR results from the murine model validated that FOS, STAT6, SP1, and SRF were key transcription factors that highly involved in anaphylaxis complicated STEMI. STAT6 is a member of the signal transducer and activator of transcription family. STAT6-deficient mice lack Th2-type allergic responses and cannot undergo class switching to produce IgE against allergens (Shimoda et al., 1996), and STAT6 gain-of-function variant exacerbates allergic inflammation (Takeuchi et al., 2023). Clinical studies indicate that two STAT6 gene variants, rs324015 and rs1059513, are significantly associated with food allergy and more severe allergic symptoms (van Ginkel et al., 2018). Moreover, STAT 6 is activated in post-infarction hearts (El-Adawi et al., 2003), in hypertrophied hearts (Mascareno et al., 1998), and in the heart subjected to ischemia/reperfusion (Mascareno et al., 2001). Disruption of STAT6 signal promotes cardiac fibrosis and impairs cardiac contractility (Hikoso et al., 2004; Zhang et al., 2020). These studies indicate that STAT6 is involved in the pathophysiology of both allergy and cardiac dysfunction. SRF, serum response factor, is a pivotal factor that not only regulates the expression of FOS, but also interacts with transcription factor SP1 (Deshpande et al., 2022). Precise regulation of SRF expression is critical for cardiac signal transduction, myocardial contractility and cytoskeletal remodeling, and transcription dysregulation of SRF leads to adverse cardiac remodeling and ultimately heart failure, suggesting the vital role played by SRF in cardiac development and disease (Li et al., 2020; Deshpande et al., 2022). FOS, STAT6, SP1, and SRF identified in our study might represent a transcriptional regulatory signature of anaphylaxis with STEMI predisposition.

Currently, many cases of anaphylaxis-related STEMI are underdiagnosed or misdiagnosed due to lack of effective detection biomarkers. The hub genes identified here constitute a whole blood gene signature shared by anaphylaxis and STEMI, and based on the logistic regression model, DUSP1 and CLEC4D are identified as blood diagnostic markers with high diagnostic efficacy, which hold a promise for diagnostic assessment of anaphylaxis complicated STEMI in clinical practice. Moreover, treatment guidelines for anaphylaxis complicated with STEMI have not been fully established. Injectable epinephrine (adrenaline) is now used as the first-line therapy for anaphylaxis (Muraro et al., 2022), yet its use in patients with allergic angina may bring adverse effects, such as ventricular arrhythmias and worsening myocardial ischemia. Meanwhile, medications used to treat STEMI, such as angiotensin-converting enzyme inhibitors and β-blockers potentially aggravate anaphylaxis (Lieberman and Simons, 2015). The adverse effects of these medications might hamper the effective management of this clinical emergency, thus novel therapeutic medications for anaphylaxis complicated STEMI are in urgent need (Lieberman and Simons, 2015). In this study, we identified four approved drugs that have the potential to be repurposed against hub genes involved in anaphylaxis complicated STEMI, including anakinra, baclofen, andecaliximab, and albuterol targeting IL1R2, FOS, MMP9, and DUSP1, respectively. Anakinra is a recombinant IL-1R antagonist approved for the treatment of COVID-19 related pneumonia and rheumatoid arthritis. A cohort study evaluating the clinical effectiveness of anakinra in patients with COVID-19 showed that anakinra had a favorable improvement in respiratory insufficiency and hyperinflammation (Cavalli et al., 2021), two pathological processes that also have pivotal roles in anaphylaxis. Besides, in the VCUART trail, administration of anakinra to patients with STEMI for 14 days significantly decreased the incidence of new-onset heart failure and heart failure hospitalization compared with placebo (Abbate et al., 2013; Abbate et al., 2020), supporting the benefits of IL-1 blockade with anakinra in slowing STEMI progression. DGIdb identified baclofen as an FDA-approved drug that potentially target FOS. Baclofen, a skeletal muscle relaxant that acts on the spinal cord nerves to reduce spasms, is commonly used in patients with multiple sclerosis. The application of baclofen in anaphylaxis and STEMI is rarely reported and deserves further study. Andecaliximab, a recombinant IgG4 monoclonal antibody targeting MMP9, is under development for the treatment different types of diseases, such as rheumatoid arthritis, Crohn’s disease, and non-small cell lung cancer (Gossage et al., 2018; Schreiber et al., 2018). Due to the critical role of MMP9 in STEMI and anaphylaxis, andecaliximab might be a promising strategy to curtail anaphylaxis complicated STEMI. According to DGIdb results, DUSP1 has a good interaction with albuterol, a β2 adrenergic agonist that relaxes muscles in the airways. Presently, albuterol is used as a second-line drug to relieve wheezing during anaphylaxis attacks (Irani and Akl, 2015). Moreover, in a randomized controlled trial, albuterol improved pulmonary vascular reserve and enhanced cardiac output reserve in patients with HFpEF (Reddy et al., 2019), showing its cardiovascular benefits. Collectively, these repurposed drug candidates provide a window of opportunity for the development of much-needed drugs for anaphylaxis complicated STEMI.

There are some limitations in the present study. First, the biological functions of immune cell subsets and hub genes shared by anaphylaxis and STEMI require further *in-vivo* and *in-vitro* experimental verification. Second, public microarray datasets for anaphylactic studies are limited, and the sample size in this study is relatively small.

## 5 Conclusion

By comprehensively profiling the peripheral whole blood transcriptome of anaphylactic and STEMI individuals, we identified the biological processes, special immune effector cell subsets, and hub genes shared by anaphylaxis and STEMI, laying down a foundation for further our mechanistic understanding of anaphylaxis complicated with STEMI. Hub genes identified here (IL1R2, FOS, MMP9, DUSP1, and CLEC4D) represent a whole blood gene signature of anaphylaxis with STEMI predisposition and provide candidate diagnostic and therapeutic targets for follow-up studies.

## Data Availability

The original contributions presented in the study are included in the article/Supplementary Material, further inquiries can be directed to the corresponding author.
