# Supplementary material for: Identification of the biological processes, immune cell landscape, and hub genes shared by acute anaphylaxis and ST-segment elevation myocardial infarction
Source: Front Pharmacol. 2023 Jul 4;14:1211332. doi: 10.3389/fphar.2023.1211332 (PMC10353022; doi:10.3389/fphar.2023.1211332)
Supplement: Supplementary file 1 [file DataSheet1.pdf]

Supplementary Material

Identification of the Biological Processes, Immune Cell Landscape, and Hub Genes Shared by Acute Anaphylaxis and ST-Segment Elevation Myocardial Infarction

Zekun Peng[1], Hong Chen[1], Miao Wang[1,2*]

*** Correspondence:** Miao Wang: miao.wang@pumc.edu.cn or wangmiao_frank@yahoo.com

Supplementary Figures and Tables

1.1 Supplementary Figures

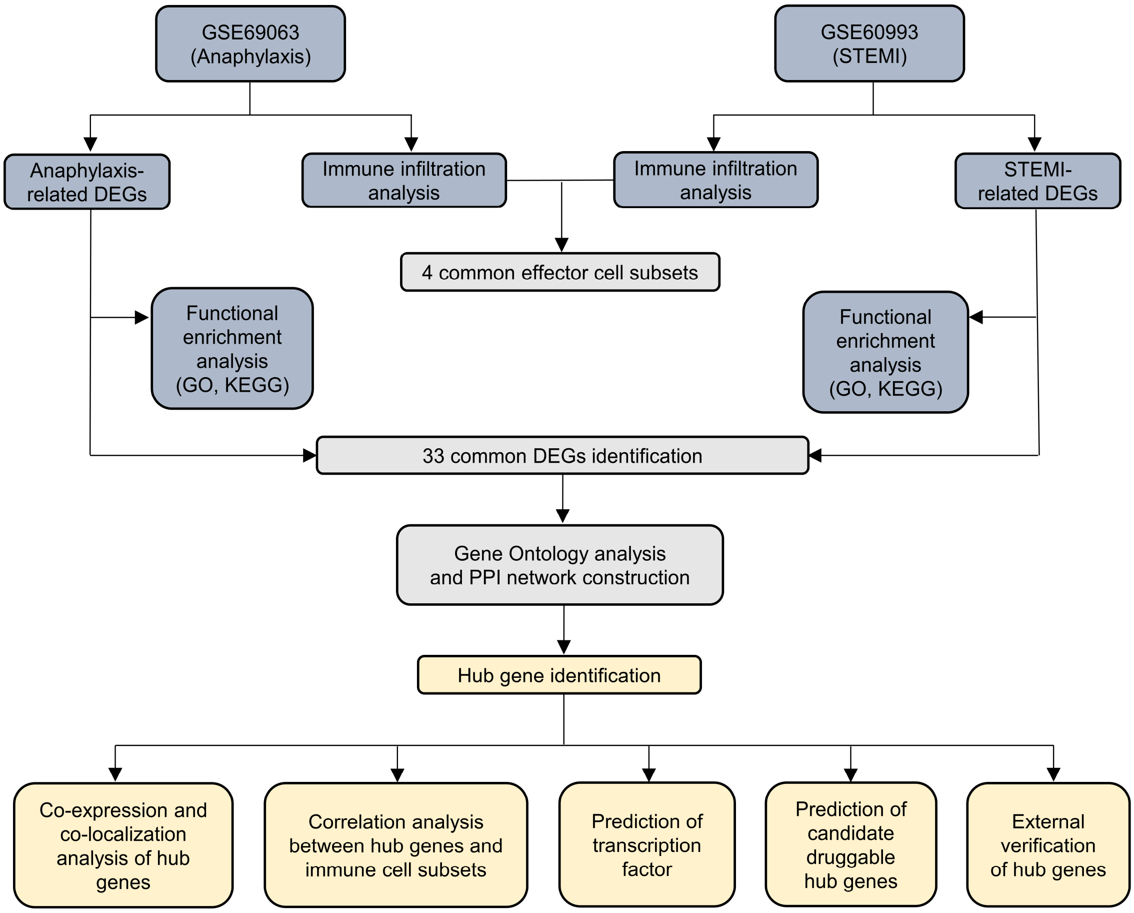

Supplementary Figure S1. Workflow of the whole study.

DEGs: differentially expressed genes; GO: gene ontology; KEGG: Kyoto encyclopedia of genes and genomes; PPI: protein-protein interaction.

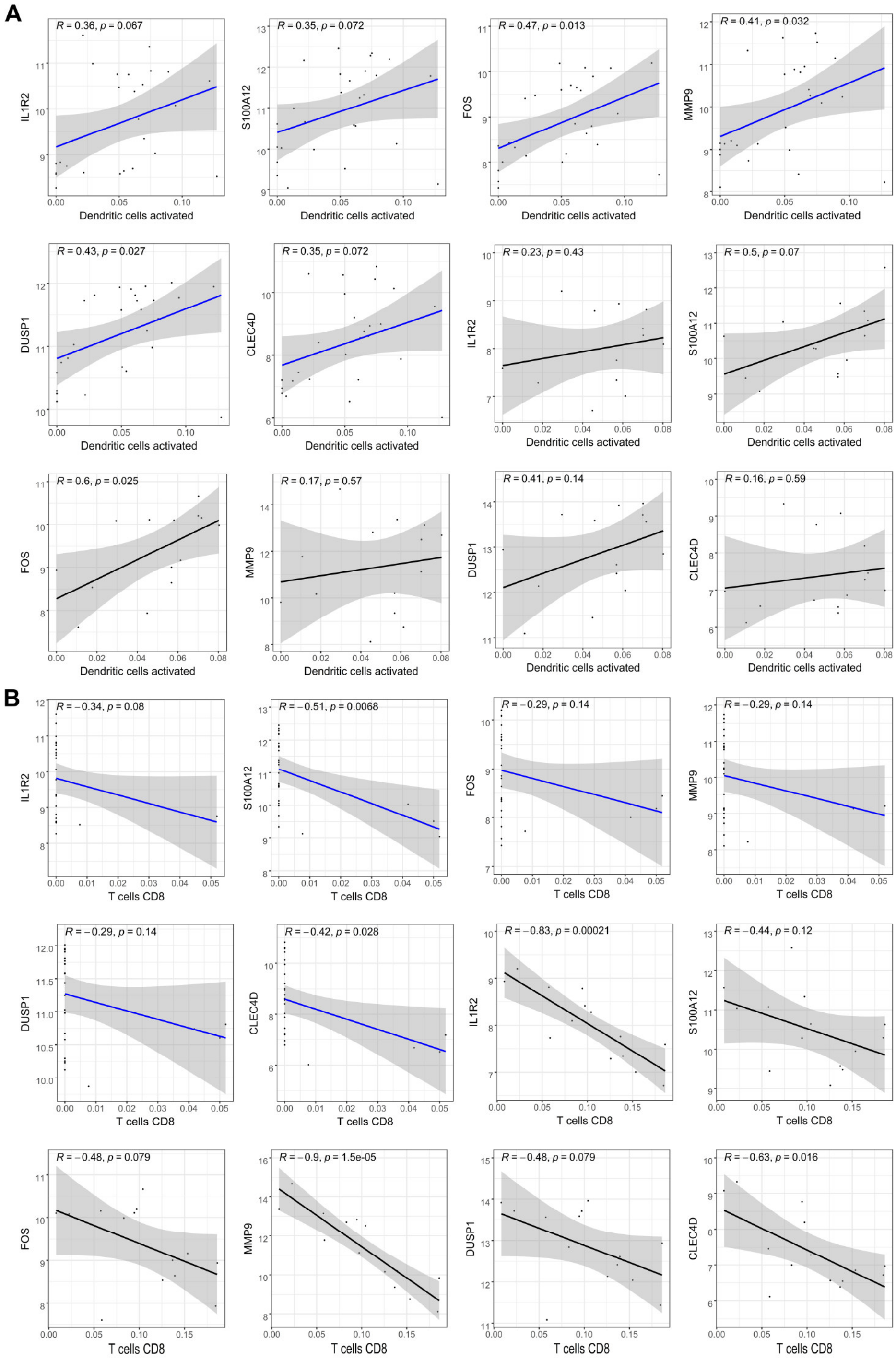

Supplementary Figure S2. Pearson's correlation analysis between hub genes and immune cells.

Scatter diagrams of the correlations between hub gene expression and immune cell abundance in anaphylactic samples (blue lines) and STEMI samples (black lines). **A,** Correlations between hub genes and activated dendritic cell. **B,** Correlations between hub genes and CD8^{+} T cell. R>0 indicates a positive correlation and R<0 indicates a negative correlation. p<0.05 was considered statistically significant.

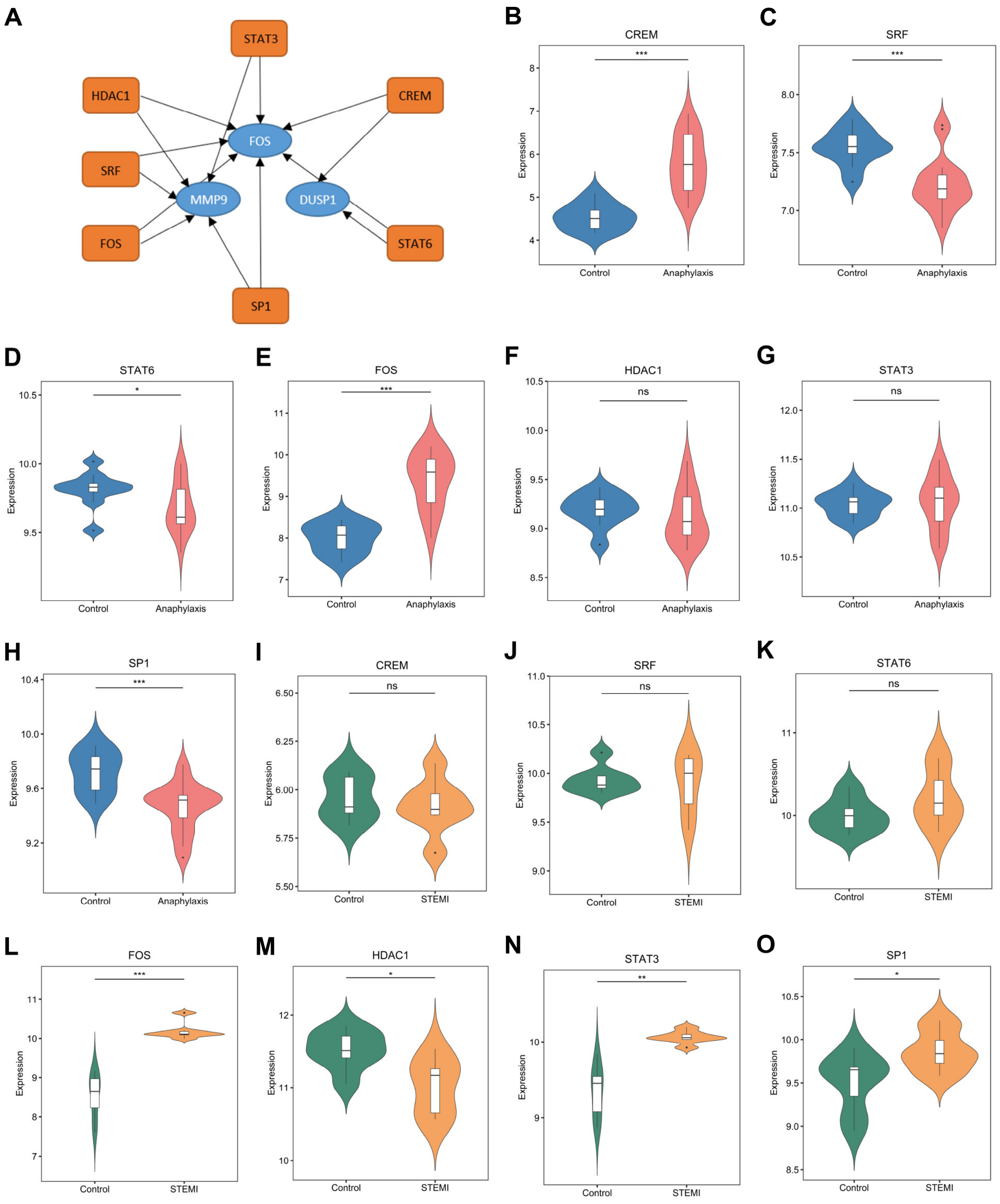

Supplementary Figure S3. Construction of transcription factor-hub gene regulatory network and verification of transcription factor expression.

A, Prediction of transcription factors via TRRUST. Orange indicates transcription factors and blue indicates hub genes. **B-H,** Validation of transcription factor expression (CREM, SRF, STAT6, FOS, HDAC1, STAT3, SP1) in GSE69063 (anaphylaxis dataset). **I-O,** Validation of transcription factor expression (CREM, SRF, STAT6, FOS, HDAC1, STAT3, SP1) in GSE60993 (STEMI dataset).

1.2 Supplementary Tables

1.1.1 Supplementary Table S1. The details of GEO datasets used in the current study

Disease	Dataset	Platform	Case (n)	Control (n)
Anaphylaxis	GSE69063	GPL19983	17	10
STEMI	GSE60993	GPL6884	7	7
Anaphylaxis	GSE47655	GPL17244	6	6
STEMI	GSE61144	GPL6106	7	10

Abbreviations: GEO, gene expression omnibus; STEMI, ST-segment elevation myocardial infarction.

1.1.2 Supplementary Table S2. The description and major functions of hub genes

Gene symbol	Description	Function
IL1R2	interleukin 1 receptor type 2	1. immune response;<br>2. cytokine-mediated signaling pathway;<br>3. negative regulation of interleukin-1 mediated signaling pathway;
S100A12	S100 calcium-binding protein A12	1. neutrophil chemotaxis;<br>2. inflammatory response;<br>3. positive regulation of I-kappa B kinase/NF-kappa B signaling;
FOS	Fos proto-oncogene, AP-1 transcription factor subunit	1. regulation of transcription from RNA polymerase II promoter;<br>2. cellular response to ROS;<br>3. inflammatory response;
MMP9	matrix metallopeptidase 9	1. Proteolysis;<br>2. extracellular matrix organization;<br>3. positive regulation of cell migration;
DUSP1	dual specificity phosphatase 1	1. inactivation of MAPK activity;<br>2. protein dephosphorylation;<br>3. response to calcium ion;

CLEC4D	C-type lectin domain family 4 member D	1. adaptive immune response;<br>2. T cell differentiation involved in immune response;<br>3. Fc-gamma receptor signaling pathway;

Abbreviations: ROS, reactive oxygen species; MAPK, mitogen-activated protein kinase.

1.1.3 Supplementary Table S3. Transcription factor-hub gene regulatory network analyzed via the TRRUST database

Transcription factor	Description	P-value	Hub gene
CREM	cAMP-responsive element modulator	7.53E-05	DUSP1, FOS
SRF	serum response factor	7.53E-05	MMP9, FOS
STAT6	signal transducer and activator of transcription 6	0.000158	FOS, DUSP1
FOS	Fos proto-oncogene	0.000397	MMP9, FOS
HDAC1	histone deacetylase 1	0.000616	MMP9, FOS
STAT3	signal transducer and activator of transcription 3	0.00243	FOS, MMP9
SP1	Sp1 transcription factor	0.0246	MMP9, FOS

Abbreviations: DUSP1, dual specificity phosphatase 1; MMP9, matrix metallopeptidase 9.

1.1.4 Supplementary Table S4. Transcription factors of hub genes predicted by the ChEA3 platform

Transcription factor	Description	Score	Hub gene	Library
FOS	Fos proto-oncogene	209.2	DUSP1, IL1R2, S100A12, FOS, MMP9	ARCHS4 Coexpression,79;<br>ENCODE ChIP-seq,8;<br>Enrichr Queries,931;<br>ReMap ChIP-seq,9;<br>GTEx Coexpression,19
STAT3	signal transducer and activator of transcription 3	289.2	DUSP1, IL1R2, S100A12, FOS, MMP9	Literature ChIP-seq,2;<br>ARCHS4 Coexpression,654;<br>ENCODE ChIP-seq,6;<br>Enrichr Queries,483;<br>ReMap ChIP-seq,11;<br>GTEx Coexpression,579

STAT6	signal transducer and activator of transcription 6	377.5	FOS	Literature ChIP-seq,144; ARCHS4 Coexpression,162; Enrichr Queries,960; GTEx Coexpression,244
SP1	Sp1 transcription factor	385.6	DUSP1, FOS	ARCHS4 Coexpression,441; ENCODE ChIP-seq,50; Enrichr Queries,1004; ReMap ChIP-seq,78; GTEx Coexpression,355
SRF	serum response factor	500	DUSP1, FOS	Literature ChIP-seq,122; ARCHS4 Coexpression,559; ENCODE ChIP-seq,16; Enrichr Queries,830; ReMap ChIP-seq,51; GTEx Coexpression,1422
CREM	cAMP-responsive element modulator	534.2	DUSP1, FOS	Literature ChIP-seq,95; ARCHS4 Coexpression,763; Enrichr Queries,466; ReMap ChIP-seq,34; GTEx Coexpression,1313

Abbreviations: DUSP1, dual specificity phosphatase 1; IL1R2, interleukin 1 receptor type 2; S100A12, S100 calcium-binding protein A12; MMP9, matrix metallopeptidase 9.
